# Measurement Models for Carbon Dioxide Emission Factors of Passenger Cars Considering Characteristics of Roads and Traffic

**DOI:** 10.3390/ijerph18041594

**Published:** 2021-02-08

**Authors:** Guoqiang Zhang, Lianghui Wu, Jun Chen

**Affiliations:** School of Transportation, Southeast University, Nanjing 210096, China; 220183083@seu.edu.cn (L.W.); chenjun@seu.edu.cn (J.C.)

**Keywords:** measurement models, carbon dioxide emission factors, passenger cars, characteristics of roads and traffic

## Abstract

In order to effectively control carbon dioxide emissions of motorized vehicles, it is very important to measure their carbon dioxide emission factors. The objective of this paper was to develop measurement models for the carbon dioxide emission factors of passenger cars. Road systems of downtown areas of four typical Chinese counties were explored and 12 types of basic road networks were recognized and defined. With PTV Vissim, microscopic traffic simulation models were set up for every type of basic road network, average speeds of the simulated cars were collected, and carbon dioxide emissions were calculated using MOVES (Motor Vehicle Emission Simulator) software. For model development, the paper put forth two compound explanatory variables: the weighted average of segment lengths and the sum of critical ratios of volume to saturation flow rate. Six functional relationships for the variables were tested and the double exponential function was proven to be the most appropriate. Finally, for each of the 12 types of basic road networks, a measurement model for carbon dioxide emission factors was calibrated using the double exponential function for the variables. The measurement models can be used to estimate the carbon dioxide emissions of passenger cars concerning potential improvement schemes impacting traffic demand and/or traffic supply.

## 1. Introduction

Although carbon dioxide has no direct impact on human health, it is, however, a major greenhouse gas emission that would trap heat in the atmosphere and cause great climate changes around the world, which would enormously threaten the existence of human lives [1]. With the fast development of the economy and society, passenger cars are becoming ordinary transportation modes for the Chinese. The trend is especially obvious for inhabitants of downtown areas of Chinese counties because public transportation is comparatively less sufficient in counties than in big cities. Therefore, it can be expected that carbon dioxide emissions in downtown areas of the counties will continue to increase substantially and there is an urgent need for the study of their measurement in order to better guide the sustainable development of Chinese counties, which has become one of the major aims of the local governments.

Compared with rural highway systems, the roadway systems of downtown areas are very complex. They are composed of numerous elements such as road segments and signalized intersections, which interact with each other and make it rather difficult to estimate the carbon dioxide emissions of motorized vehicles. Furthermore, many factors concerning the characteristics of road and traffic impact vehicular movements are as follows: the length of road segments, the number of lanes of road segments and intersection approaches, shapes of intersections, types of motorized vehicles, traffic volumes of road segments, distribution of traffic volumes among different lanes of road segments and length, age and desired speed of motorized vehicles, and so on. Since vehicular movements have a close relationship with carbon dioxide emissions, the above factors may also impact carbon dioxide emissions. How to take the various impacting factors into consideration is also a great challenge. As passenger cars are the predominant component of motorized vehicles in downtown areas, the study was mainly concerned with their carbon dioxide emissions.

Against this backdrop, the general objective of this paper was to put forth a series of measurement models for the carbon dioxide emission factors of passenger cars in downtown areas of typical Chinese counties, taking into consideration the characteristics of roads and traffic. To be specific, it included the following goals: (1) designing a flowchart of the research framework, which help to organize different aspects of the research and make it more efficient to carry out various tasks; (2) decomposing road networks into a series of basic road networks, which are more suitable for traffic simulation and model development, based upon the analysis of the characteristics of roads in the downtown areas of Chinese counties; and (3) developing measurement models for carbon dioxide emission factors based upon the traffic simulation of basic road networks.

## 2. Literature Review

Carbon dioxide emissions of motorized vehicles can be measured through lab tests using relevant lab test equipment, or through field measurements using portable emissions measurement systems [2,3,4]. Vehicle emissions can also be estimated by various emissions models and approaches. The International Vehicle Emission (IVE) model developed by joint researchers from the International Sustainable System Research Center and the University of California, Riverside, uses vehicle technology distributions, power-based driving factors, vehicle soak distributions, and meteorological factors to estimate emissions [5]. The model has been applied widely to evaluate various emissions of motorized vehicles and analyze all kinds of measures concerning such emissions [6]. Motor Vehicle Emission Simulator (MOVES), developed by the U.S. Environmental Protection Agency, uses the average speed distributions of motorized vehicles to estimate their emissions [7]. Such estimation models and approaches provide convenient methods for various researches concerning the emissions of motorized vehicles.

There are many factors impacting the emissions of motorized vehicles such as vehicle characteristics, vehicle operational activity, fuel types, pavement characteristics, the layouts of road networks, geometric design of roads or streets, traffic volumes, signal control plans, and so on. Research has been carried out to explore the influence of some of the factors. Choi et al. studied the impacts of high speed and constant speed, based on vehicle-specific power-based modal models for light duty gasoline vehicles, using data from portable emission measurement systems [8]. Ghafghazi et al. developed a microscopic traffic simulation and emission modeling system, aiming at quantifying the effects of different types of traffic calming measures on vehicle emissions both at a link-level and at a network-level [9].

Abou-Senna et al. explored the carbon dioxide emissions of motorized vehicles on limited access highways in a microscopic and stochastic environment using an optimal design approach and speed was found to have a significant impact [10]. Vlieger et al. studied the influence of driving behavior and traffic conditions on fuel consumption and emissions for a small test fleet of passenger cars; city traffic was found to have the highest fuel consumption and emissions [11]. Gu et al. investigated the traffic-related emission impacts of work zones using an urban freeway case study; a VISSIM test bed combined with the Environmental Protection Agency’s MOVES emission model was used to estimate the total emissions assuming daytime and nighttime lane-closure scenarios [12]. You et al. explored the relationship between the horizontal curve radius and carbon dioxide emissions on freeways from field tests under real world driving conditions through the development of an emission factor model (EFM) [13]. Li et al. studied the impacts of weaving segment configuration on vehicle emissions, identified important predictors for emission estimations, and developed a nonlinear normalized emission factor (NEF) model for weaving segments [14].

Li et al. and Jiao built evaluation models to estimate vehicle emission factors with data concerning pavement conditions and found that the relationship between roadway pavement conditions and vehicle emissions was non-linear [15,16]. Peters et al. explored approaches as to how to coordinate traffic signals to reduce congestion and carbon dioxide emissions [17]. Papson et al. analyzed vehicle emissions at congested and uncongested signalized intersections under three traffic intersection scenarios and found that the emissions were much less sensitive to congestion than control delay [18]. Xu et al. developed emission-specific VSP bins for estimating carbon dioxide (CO_2_) emissions for light-duty vehicles by using the real world data collected in Beijing [19]. Yao et al. investigated the characteristics of vehicle specific power (VSP) distributions and tested the speed, acceleration, and grade—the three most critical variables for VSP for light duty vehicles [20,21].

Although many studies have estimated the carbon dioxide emissions of motorized vehicles for various roads or streets and have established estimation models for some specific impacting factors, there are, however, quite a few studies that have aimed at the analysis of more complicated transportation systems such as urban road networks and have developed estimation models with more comprehensive explanatory variables. Furthermore, compared with metropolitan areas of big cities, downtown areas of Chinese counties have received little attention, despite the fact that they are numerous and their total carbon dioxide emissions of motorized vehicles are also very large.

## 3. Methodology

The proposed method is to develop regression models for carbon dioxide emission factors of passenger cars in various kinds of road networks. According to real road data, microscopic traffic simulation was performed to obtain the average travel speed distributions of simulated passenger vehicles under various schemes, based upon which carbon dioxide emission factors were then calculated to provide data for model construction and calibration.

### 3.1. Research Framework

**Figure 1:**
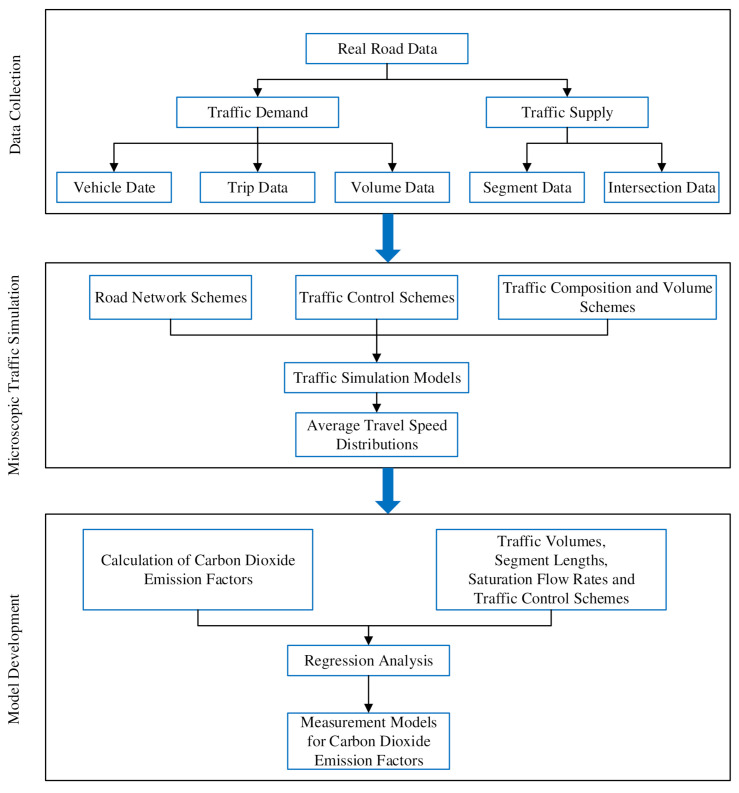
Flowchart of the research framework.

Figure 1 is the flowchart of the research framework, which includes three steps: (1) data collection; (2) microscopic traffic simulation; and (3) model development. The first step “data collection” is to obtain all kinds of data concerning traffic demand and traffic supply, analysis of which provides the basic information for microscopic traffic simulation. Data concerning traffic demand such as the characteristics of passenger cars, spatial and temporal distributions of trips, and thee volumes of segments and intersections are both myriad and variable. They take much more effort in data collection and need to be obtained through many sources. Data concerning traffic supply such as the geometric designs of segments and intersections are relatively simple and can be easily collected.

The second step of “microscopic traffic simulation” is to construct microscopic traffic simulation models for various schemes of road network, traffic control, traffic composition and volume, which are based on the data of real roads. The simulation models are implemented, and data concerning the travel time of vehicles from the simulations are then collected for the calculation of the average travel speed distributions. The third step “model development” is based upon data from the aforementioned two steps. Using average travel speed distributions for various simulation schemes, carbon dioxide emission factors can then be calculated, and with the addition of data concerning traffic volumes, segment lengths, saturation flow rates, and traffic control schemes, regression analysis is carried out and measurement models for carbon dioxide emission factors are constructed and calibrated. Figure 1 sketches the outline for the research and achieved the first specific goal of the paper.

### 3.2. Microscopic Traffic Simulation

Using the traffic microsimulation software PTV Vissim (PTV Planung Transport Verkehr AG, Karlsruhe, Germany) [22], microscopic traffic simulation models can be constructed to simulate real road systems. The data of real roads are analyzed to calibrate some important parameters of the simulations such as free flow speed distribution and vehicle length distribution. Based upon real road data, various layouts and geometric designs of the real roads can be simulated. Possible traffic compositions and volumes for traffic simulation can also be taken into consideration. When road network and traffic compositions and volumes for a simulation model are decided, the appropriate traffic control plan will then be figured out and simulated. Specifically, for signalized control, a phase plan can be developed according to the traffic volumes of different lane groups, and cycle length can be calculated as follows:

(1) $$C=\frac{LX}{X-\sum_{i=1}^{N}Y_{i}}.$$

where C indicates the cycle length of a phase plan, (s); *L* indicates the total lost time per cycle, (s); *X* indicates the desired ratio of volume to capacity for the intersection; *Y_i_* indicates the critical ratio of volume to saturation flow rate for the ith phase; and *N* indicates the number of phases of a phase plan.

When the cycle length of a phase plan is calculated using Equation (1), it will be assigned to each phase according to the volumes and saturation flow rates of the critical lane groups. The green time for each phase can thus be figured out.

When the traffic simulation models are constructed, they are run and data concerning the travel time of the simulated passenger cars are collected to obtain the average travel speeds, based upon which the carbon dioxide emissions can be calculated. Given that during the process of simulation, simulated vehicles appear in the system randomly, resulting in a shorter or longer delay, the running of each simulation will last more than one hour to accumulate enough data to smooth out the effect of randomness.

### 3.3. Calculation of Carbon Dioxide Emission Factors

With data concerning the distributions of the average travel speed from microscopic traffic simulation, as discussed above, the carbon dioxide emission factors can be calculated by using the MOVES (the motor vehicle emission simulator) [23] modeling software of the U.S. Environmental Protection Agency. MOVES is a database driven software with a disaggregate emission algorithm that provides flexibility for both inputs and outputs in the emission estimation process. The MOVES model calculates the emission factors by determining the vehicle operating mode, which is based on speed, acceleration, and vehicle specific power (VSP):

(2) $$\mathrm{VSP}=\frac{Av_{t}+Bv_{t}^{2}+Cv_{t}^{3}+mv_{t}a_{t}}{m},$$

where *v_t_* indicates the speed at time *t* (m/s); *a_t_* indicates the acceleration at time *t* (m/s^2^); *m* indicates the mass of the vehicle, usually referred as “weight” (tons); and *A*, *B*, and *C* indicate the track-road coefficients, representing rolling resistance, rotational resistance and aerodynamic drag, respectively.

By providing the average travel speed and road characteristics, a default driving cycle is assigned that best fits the given data, and provides an emission factor associated with the distribution of average travel speed. Total emissions are then calculated by multiplying the emission factor with vehicle kilometers traveled. Aside from vehicle activities, MOVES requires other input data such as source type, model year, calendar year, temperature, and humidity.

### 3.4. Regression Analysis

For a specific kind of road network, factors such as the lengths of segments and volumes of lanes have a strong influence on the carbon dioxide emissions of passenger cars. By regression analysis, it is possible to explore the impacting factors systematically and accurately, and set up measurement models for carbon dioxide emission factors. As there are various impacting factors involved in the research, multiple regression analysis can be used and its simplest kind is the multiple linear regression model, which can be written in the population as follows:

(3) $$y=\beta_{0}+\beta_{1}x_{1}+\beta_{2}x_{2}+\cdots +\beta_{k}x_{k}+\mu ,$$

where y indicates the dependent variable; $x_{i}$ indicates the independent variable (i=1,2,⋯,k); $\beta_{0}$ is the intercept; $\beta_{1}$ is the parameter associated with the independent variable $x_{1}$; $\beta_{2}$ is the parameter associated with independent variable $x_{2}$, and so on; and μ is the error term representing factors other than $x_{i}\left(i=1,2,\cdots ,k\right)$ that affect y.

When relationships between the dependent variable and all the independent variables are not linear, non-linear regression analysis are considered. In order to ensure that the appropriate relationship is accepted, various relationships are used, and their model goodness-of-fit (GOF) is studied and the best one shall thus be decided. The commonly used measure of model GOF is the R-squared statistic, which is as follows:

(4) $$R^{2}=1-\frac{SSE}{SST},$$

where *SSE* is the sum of square errors and *SST* is the total sum of squares.

*R*^2^ is commonly interpreted as the proportion of total variance explained by independent variables. When *SSE* equals zero, *R*^2^ is 1, and all of the variance is explained by the model. *R*^2^ ranges between 0 and 1, and a bigger value indicates whether its corresponding relationship is more appropriate. Therefore, comparing the values of *R*^2^ for different relationships of the variables can help to choose the best model. Furthermore, the Akaike information criterion (AIC) and the Bayesian information criterion (BIC) are often used for model selection, and for these two criteria, a smaller value indicates better model performance.

## 4. Data Collection

For the purpose of setting up representative traffic simulation models, data collection was carried out in four Chinese counties, which lie in different districts of China and have typical roadway systems in downtown areas. The four counties under investigation were Changxing County, Qingcheng County, Jintang County, and Wuan County.

Changxing County lies in the east of China, by Taihu Lake, near Shanghai City. It represents regions in the east part of China near the Pacific Ocean, which is populous and the economy is prosperous. Qingcheng County lies in the northwest of China in a mountainous area. It represents the mountainous northwestern regions of China, where climates are very harsh and populations are thinly distributed. Jintang County lies in the southwest of China on a fertile plain. It represents the southwestern regions of China, where it is fairly populous and the economy is developing very quickly. Wuan County lies in the northeastern regions of China, where the iron and steel industry is very advanced and the air quality is worse during the winter seasons. Since the four counties lie far apart in geography and can represent different parts of China, data collected from their road systems are representative of Chinese counties.

**Figure 2:**
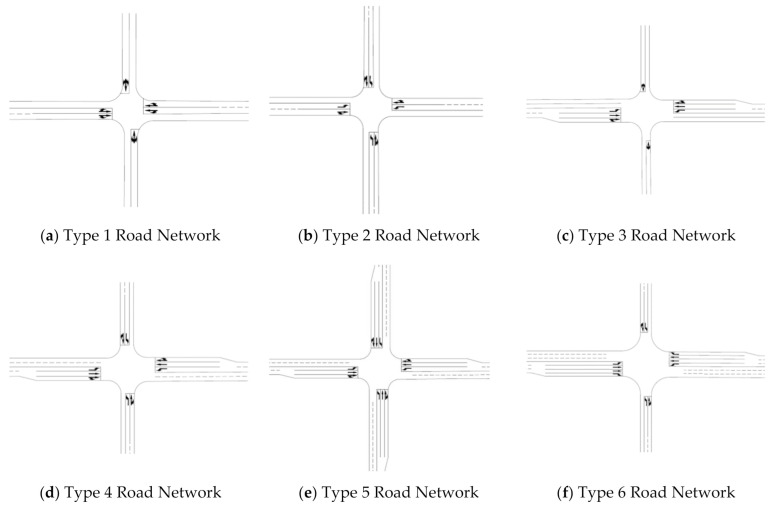

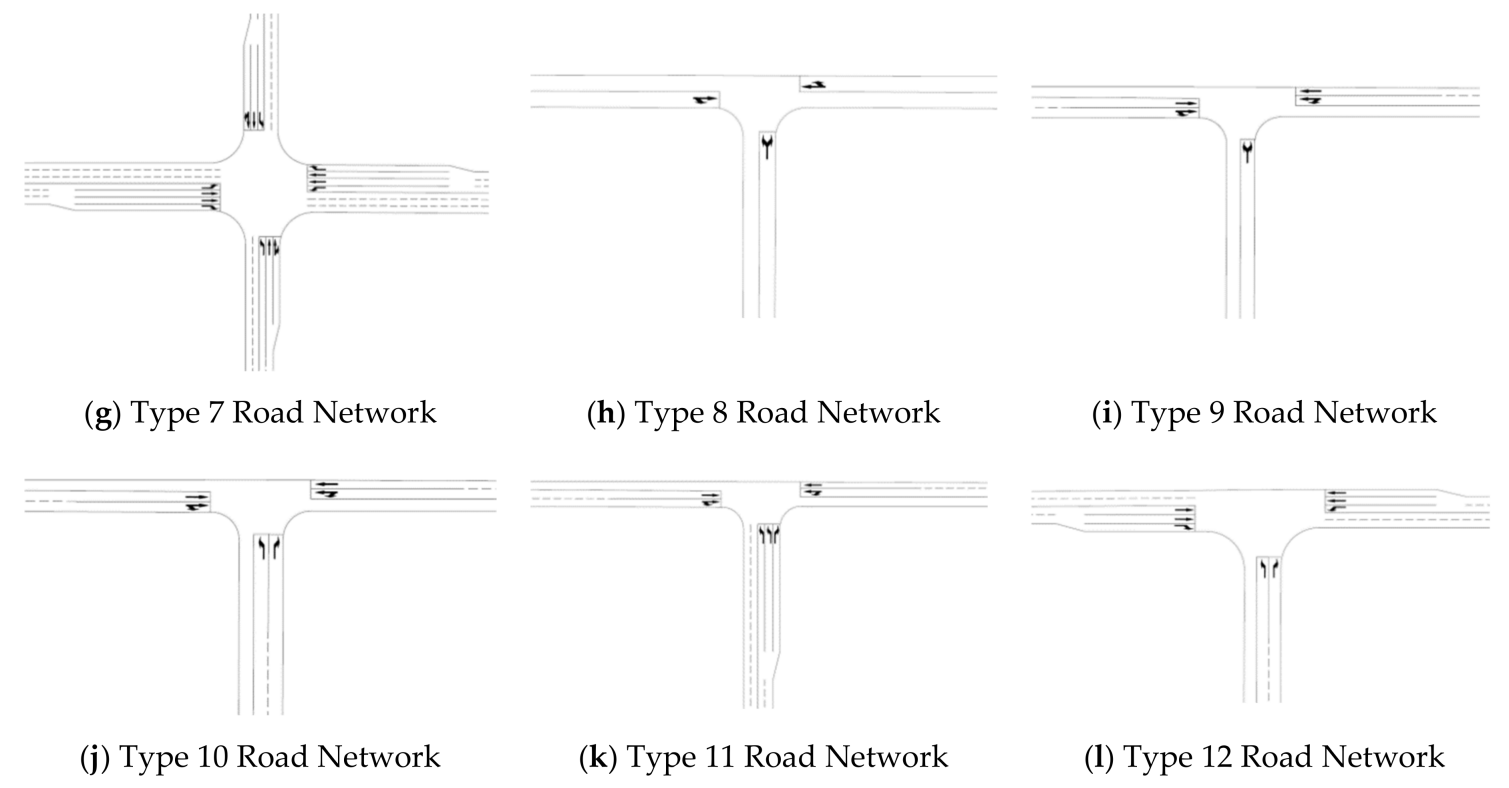
Definitions for twelve types of basic road networks. (**a**): Type 1 Road Network, (**b**): Type 2 Road Network, (**c**): Type 3 Road Network, (**d**): Type 4 Road Network, (**e**): Type 5 Road Network, (**f**): Type 6 Road Network, (**g**): Type 7 Road Network, (**h**): Type 8 Road Network, (**i**): Type 9 Road Network, (**j**): Type 10 Road Network, (**k**): Type 11 Road Network, (**l**): Type 12 Road Network.

In the study, only arterial and collector roads (or streets) were taken into consideration. Characteristics of roads (or streets) are one of the most important factors that have great influence upon the carbon dioxide emissions of motorized vehicles. Road segments are directional and are defined as road elements that start from the stop line of upstream intersection and end at the stop line of downstream intersection. Urban road networks can be decomposed into signalized intersections and their adjacent segments, which bring various vehicles to the intersections. Such signalized intersections and their adjacent segments can be regarded as a basic form of urban road networks. Road networks in downtown areas of the four counties discussed above were decomposed and analyzed and 12 types of basic road networks were recognized as the most common ones based upon the characteristics of the roads. Their definitions are illustrated in Figure 2, which show the achievement of the second specific goal of the paper.

The twelve types of road networks were defined according to the layouts of the road networks, number of lanes of adjacent segments connected to the intersection, and whether one or two lanes were added to approaches of the intersection compared with their upstream segments. From type 1 to type 7, the layouts of road networks were similar in that they were shaped like a cross with four adjacent segments sending vehicles to the intersection. From type 8 to type 12, the layouts of road networks were t shaped, with three adjacent segments sending vehicles to the intersection.

Thee type 1 road network is cross shaped with the two segments of the major road having two lanes and the two segments of the minor road having one lane. Furthermore, no lanes are added to each of the four approaches of the intersection. The type 2 road network has the same characteristics as the type 1 road network except that the minor road segments have two lanes instead of one lane. The type 3 road network has the same characteristics as the type 1 road network except that one lane is added to either of the two opposing major road approaches of the intersection. The type 4 road network has the same characteristics as the type 3 road network except that the two minor road segments have two lanes instead of one lane. The type 5 road network has the same characteristics as the type 4 road network except that one lane is added to either of the two opposing minor road approaches of the intersection.

Being cross shaped, the type 6 road network has three lanes on either of the two segments of the major road and one lane is added to either of the two major road approaches; either of the two segments of the minor road has two lanes with no lanes added to either of the two minor road approaches. The type 7 road network has the same characteristics as the type 6 road network except that one lane is added to either of the two opposing minor road approaches of the intersection.

The type 8 road network is t-shaped with the two segments of the major road having one lane and the segment of the minor road having one lane too. Furthermore, no lanes are added to each of the three approaches of the intersection. The type 9 road network has the same characteristics as the type 8 road network except that the two segments of the major road have two lanes instead of one lane. Being t-shaped, the type 10 road network has two lanes on either of the two segments of the major road and also has two lanes on the segment of the minor road, with no lanes added to each of the three approaches of the intersection. The type 11 and type 12 road networks have the same characteristics as the type 10 road network except that type 11 has one lane added to the approach of the minor road and type 12 has one lane added to either of the two approaches of the major road.

**Figure 3:**
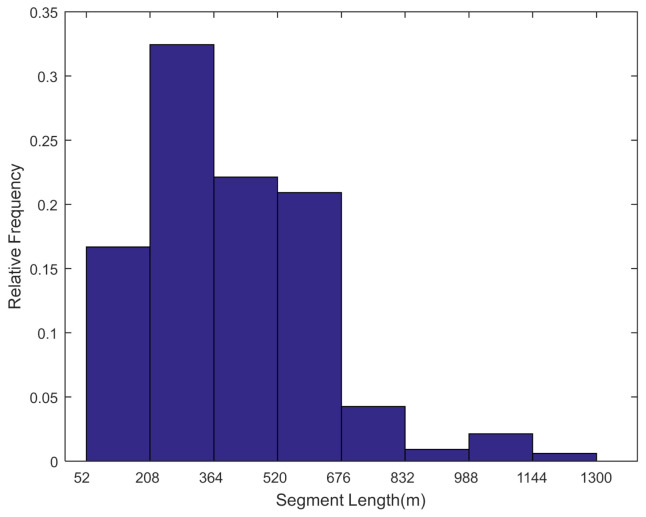
Histogram for the road segment length distribution.

Segment lengths of road networks were measured with Baidu maps on the Internet and data concerning 330 road segments were collected. The histogram for road segment lengths is illustrated in Figure 3. It indicates that most of the road segment lengths were short or moderate and 92.1% of the segment lengths ranged from 52 to 676 m. About 16.7% of segment lengths were rather short and ranged between 50 and 210 m. Only a very small portion of road segments were rather long and about 2.7% of segments were longer than 1000 m.

**Figure 4:**
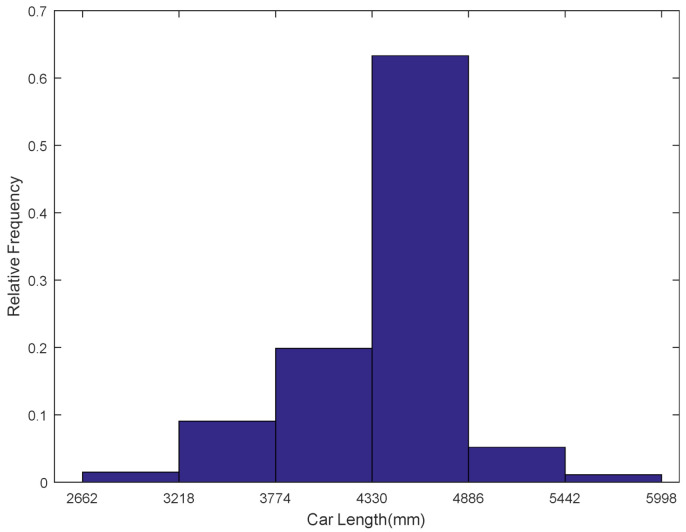
Histogram for the length distribution of passenger cars.

**Figure 5:**
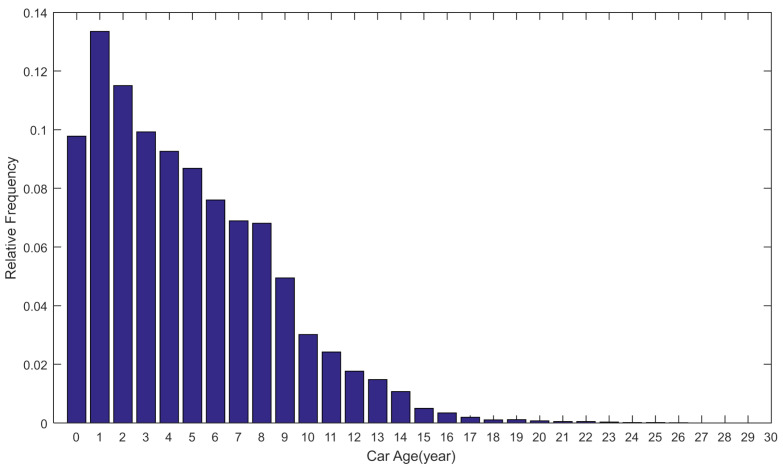
Histogram for the age distribution of passenger cars.

The lengths and ages of nearly three hundred thousand passenger cars were collected from databases of vehicle management systems, which were set up and managed by local police departments. Histograms for length distribution and age distribution are illustrated in Figure 4 and Figure 5, respectively. For length distribution, 63.31% of passenger car lengths ranged from 4330 to 4886 mm; about 97.4% of passenger car lengths were between 3218 and 5442 mm. The ages of passenger cars ranged between 0 and 29 years. Percentage of the age group between 1 and 2 years was the largest and from then on, the percentages of age groups decreased gradually with the increase in car ages. A total of 53.82% of passenger cars were less than five years, 88.74% of passenger cars were less than 10 years, and 98.49% of passenger cars were less than 15 years old.

**Figure 6:**
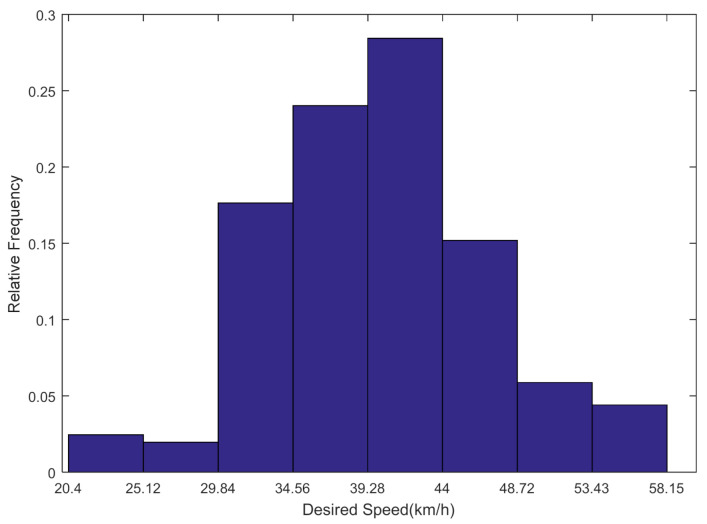
Histogram for the desired speed distribution of passenger cars.

Aided by video recorders, the desired speeds of passenger cars were measured on the roads of downtown areas of the counties by way of measuring the travel time of cars along a road segment when traffic volumes were low and drivers could choose speeds freely. In all, 204 speeds were identified as free flow speeds. The histogram for desired speed distribution is illustrated in Figure 6 where 28.43% of the desired speeds ranged from 39.3 to 44 km/h and 85.2% of desired speeds were in the range of 29.8 to 48.7 km/h.

Data concerning road networks and road segment lengths were collected to help to set up simulation road networks. Data concerning the lengths of passenger cars and their desired speeds were used for the calibration of traffic simulation models. Data concerning the age of passenger cars were used to calculate the carbon dioxide emission factors. Furthermore, other data such as those concerning the trips of inhabitants and traffic volumes of road segments and intersections were also collected. A questionnaire survey was carried out to obtain information concerning the characteristics of various trips of inhabitants in downtown areas of the counties. It was found that commuting trips accounted for more than 80% of all trips and more than twenty percent of the trips were performed by passenger cars. Data from the monitoring systems of road networks were combined with data collected from the roads by the research team and data concerning the GPS trajectories of motorized vehicles to explore the characteristics of traffic volumes. Analysis indicated that passenger cars were the predominant vehicle type in the downtown areas of the counties. Unlike big cities, traffic volumes were usually at a moderate level and serious traffic congestions were very rare, even during rush hours.

## 5. Traffic Simulation and CO_2_ Emissions Calculation

According to the data analysis of the road systems discussed above, there were twelve types of basic road networks. For each of them, a microscopic traffic simulation model was developed to simulate its characteristics with PTV Vissim software. In order to explore the impacts of segment lengths and traffic volumes upon travel speeds and the carbon dioxide emissions of passenger cars for each simulation model, the corresponding simulation parameters were changed to simulate various situations that may exist in the real world, and the average travel speeds of simulated vehicles under these situations were obtained for the calculation of carbon dioxide emission factors by using MOVES.

To calibrate MOVES, research was carried out based upon the real-world local fuel consumption data from Xiaoxiongyouhao, a smartphone application aimed at processing personal actual vehicle fuel consumption according to the refueling data since 2008 [24]. Results of the research indicated that in general, a correction factor of 0.914989 can be applied for the calculation of the carbon dioxide emissions of passenger cars in the four Chinese counties. The correction factor was used in this study.

As random seeds have some impact upon the travel speeds of simulated vehicles, a test was performed to study their influence upon the travel speed distribution of passenger cars and their carbon dioxide emission factors [25]. For this purpose, a specific simulation model was run twice with two different random seeds while keeping all the other parameters unchanged. Histograms of travel speeds for the two random seeds were very much alike, meaning that they might have come from the same population. The Mann–Whitney–Wilcoxon test was performed and their respective carbon dioxide emission factors were calculated. Results indicated that influence of different random seeds upon travel speed distributions and their carbon dioxide emission factors was very insignificant. Therefore, in the study, where the carbon dioxide emissions of vehicles was the focus of research, the impact of random seeds was ignored.

For the type 1 road network, 18 different schemes concerning various road segment lengths, traffic volumes, and signal control plans were simulated. The corresponding distributions of the average travel speeds of passenger cars from traffic simulations were analyzed. Based upon the distributions, corresponding carbon dioxide emission factors were calculated by MOVES software. As a quick summary, road segment lengths for the type 1 road network ranged from 200 to 1100 m, with the mean being 650 m and the standard deviation being 333 m. Total volumes of the intersection ranged from 905 to 3424 veh/h, with the mean being 1976 veh/h and the standard deviation being 789 veh/h. Carbon dioxide emission factors ranged from 218 to 277 g/km, with the mean being 237 g/km and the standard deviation being 17 g/km.

For the type 2 road network, 73 different schemes were simulated and the corresponding carbon dioxide emission factors were calculated. As a quick summary, road segment lengths ranged from 200 to 1000 m, with the mean being 600 m and the standard deviation being 244 m. Total volumes of the intersection ranged from 847 to 3551 veh/h, with the mean being 1851 veh/h and the standard deviation being 559 veh/h. Carbon dioxide emission factors ranged from 218 to 496 g/km, with the mean being 245 g/km and the standard deviation being 46 g/km. For the type 3 road network, 24 different schemes were simulated and the corresponding carbon dioxide emission factors were calculated. As a quick summary, road segment lengths ranged from 200 to 1000 m, with the mean being 650 m and the standard deviation being 332 m. Total volumes of the intersection ranged from 1084 to 3496 veh/h, with the mean being 2240 veh/h and the standard deviation being 702 veh/h. Carbon dioxide emission factors ranged from 219 to 325 g/km, with the mean being 247 g/km and the standard deviation being 30 g/km.

For the type 4 road network, 63 different schemes were simulated and corresponding carbon dioxide emission factors were calculated. As a quick summary, road segment lengths ranged from 200 to 1000 m, with the mean being 595 m and the standard deviation being 222 m. Total volumes of the intersection ranged from 1044 to 4005 veh/h, with the mean being 2199 veh/h and the standard deviation being 623 veh/h. Carbon dioxide emission factors ranged from 216 to 358 g/km, with the mean being 238 g/km and the standard deviation being 22 g/km. For the type 5 road network, 24 different schemes were simulated and the corresponding carbon dioxide emission factors were calculated. As a quick summary, road segment lengths ranged from 200 to 1000 m, with the mean being 617 m and the standard deviation being 293 m. Total volumes of the intersection ranged from 846 to 4948 veh/h, with the mean being 2857 veh/h and the standard deviation being 1206 veh/h. Carbon dioxide emission factors ranged from 213 to 478 g/km, with the mean being 277 g/km and the standard deviation being 80 g/km. For the type 6 road network, 27 different schemes were simulated and the corresponding carbon dioxide emission factors were calculated. As a quick summary, road segment lengths ranged from 200 to 1100 m, with the mean being 650 m and the standard deviation being 332 m. Total volumes of the intersection ranged from 1197 to 5083 veh/h, with the mean being 2826 veh/h and the standard deviation being 995 veh/h. Carbon dioxide emission factors ranged from 215 to 572 g/km, with the mean being 270 g/km and the standard deviation being 88 g/km.

For the type 7 road network, 27 different schemes were simulated and corresponding carbon dioxide emission factors were calculated. As a quick summary, road segment lengths ranged from 200 to 1100 m, with the mean being 650 m and the standard deviation being 332 m. Total volumes of the intersection ranged from 1102 to 5611 veh/h, with the mean being 3571 veh/h and the standard deviation being 1212 veh/h. Carbon dioxide emission factors ranged from 211 to 447 g/km, with the mean being 268 g/km and the standard deviation being 67 g/km. For the type 8 road network, 21 different schemes were simulated and the corresponding carbon dioxide emission factors were calculated. As a quick summary, road segment lengths ranged from 200 to 1000 m, with the mean being 633 m and the standard deviation being 293 m. Total volumes of the intersection ranged from 417 to 1602 veh/h, with the mean being 1085 veh/h and the standard deviation being 313 veh/h. Carbon dioxide emission factors ranged from 223 to 285 g/km, with the mean being 237 g/km and the standard deviation being 15 g/km. For the type 9 road network, 18 different schemes were simulated and the corresponding carbon dioxide emission factors were calculated. As a quick summary, road segment lengths ranged from 200 to 1000 m, with the mean being 644 m and the standard deviation being 293 m. Total volumes of the intersection ranged from 622 to 3116 veh/h, with the mean being 1618 veh/h and the standard deviation being 656 veh/h. Carbon dioxide emission factors ranged from 214 to 272 g/km, with the mean being 232 g/km and the standard deviation being 17 g/km.

For the type 10 road network, 18 different schemes were simulated and corresponding carbon dioxide emission factors were calculated. As a quick summary, road segment lengths ranged from 200 to 1000 m, with the mean being 633 m and the standard deviation being 293 m. Total volumes of the intersection ranged from 572 to 2830 veh/h, with the mean being 1686 veh/h and the standard deviation being 665 veh/h. Carbon dioxide emission factors ranged from 210 to 245 g/km, with the mean being 219 g/km and the standard deviation being 8 g/km. For the type 11 road network, 18 different schemes were simulated and the corresponding carbon dioxide emission factors were calculated. As a quick summary, road segment lengths ranged from 200 to 1000 m, with the mean being 633 m and the standard deviation being 293 m. Total volumes of the intersection ranged from 806 to 3244 veh/h, with the mean being 1990 veh/h and the standard deviation being 619 veh/h. Carbon dioxide emission factors ranged from 215 to 228 g/km, with the mean being 221 g/km and the standard deviation being 5 g/km. For the type 12 road network, 24 different schemes were simulated and the corresponding carbon dioxide emission factors were calculated. As a quick summary, road segment lengths ranged from 200 to 1000 m, with the mean being 633 m and the standard deviation being 293 m. Total volumes of the intersection ranged from 606 to 3468 veh/h, with the mean being 2138 veh/h and the standard deviation being 802 veh/h. Carbon dioxide emission factors ranged from 212 to 288 g/km, with the mean being 232 g/km and the standard deviation being 19 g/km.

## 6. Model Development

With these data concerning the road networks, traffic volumes, and carbon dioxide emission factors discussed above, research was carried out for the purpose of the development of measurement models for carbon dioxide emission factors by regression analysis. In order to improve the performance of measurement models and use more comprehensive information for evaluation, the research team put forth two compound variables: the weighted average of segment lengths and the sum of the critical ratios of volume to saturation flow rate. Weighted average of segment lengths can be calculated as follows:

(5) $$L_{A}=\frac{\sum_{i=1}^{N}q_{i}l_{i}}{\sum_{i=1}^{n}q_{i}},$$

where *q_i_* is traffic volume of the ith segment, which enters the intersection (veh/h); *l_i_* is the length of the ith segment (meters); and *N* is the number of segments connected to the intersection.

The sum of critical ratios of volume to saturation flow rate can be calculated as follows:

(6) $$Y=\sum_{i=1}^{M}Y_{i},$$

where *Y_i_* is the critical ratio of volume to saturation flow rate for the ith phase and *M* is the number of phases of a phase plan.

The critical ratio of volume to saturation flow rate for the ith phase is given as follows:

(7) $$Y_{i}=\frac{vol_{i}}{sat_{i}}\left(i=1,2,\cdots ,M\right),$$

where *vol_i_* is the traffic volume of the critical lane group for the ith phase (veh/h) and *sat_i_* is the saturation flow rate of the critical lane group for the ith phase (veh/h).

Before the measurement models were developed, associations between either of the two aforementioned variables and carbon dioxide emission factors were tested by the Pearson product-moment correlation coefficient based upon data from the traffic simulation and calculation of carbon dioxide emissions for the type 4 road network. The analysis indicated that there was a very strong positive linear relationship between the sum of critical ratios of volume to saturation flow rate and carbon dioxide emission factors, with the Pearson correlation coefficient being 0.755 and *p*-value being 0.000. Furthermore, there was a strong negative linear relationship between the weighted average of segment lengths and carbon dioxide emission factors, with a Pearson correlation coefficient being −0.347 and *p*-value being 0.005. The tests indicated that it was possible to develop a measurement model for the carbon dioxide emission factors with the weighted average of segment lengths and sum of the critical ratios of volume to saturation flow rate being the independent variables. The measurement model can be written as follows:

(8) $$CF=f\left(L_{A},Y\right),$$

where *CF* indicates the carbon dioxide emission factor (g/km) and f indicates the functional relationship between the independent variables and dependent variable.

In order to find an appropriate functional relationship for the variables, various models were developed with data for the type 4 road network and a comparison between different models was carried out with the R-squared statistic, AIC (the Akaike information criterion) and BIC (the Bayesian information criterion), respectively. The results are listed in Table 1. It is obvious that measurement model 4 and measurement model 6 had bigger values of R^2^ and smaller values of both AIC and BIC, which means that they are more suitable for model development. Due to its relative simplicity, model 4 and the double exponential function was finally selected in the study.

For the twelve types of road networks, measurement models for the carbon dioxide emission factors are given as follows:

(9) $$CF=a_{i}\cdot \exp\left(m_{i}\cdot L_{A}\right)+b_{i}\cdot \exp\left(n_{i}\cdot Y\right)+c_{i}\left(i=1,2,\cdots ,12\right),$$

where *a_i_*, *m_i_*, *b_i_*, *n_i_*, and *c_i_* are the parameters of the ith measurement model for the ith type of road network.

**Figure 7:**
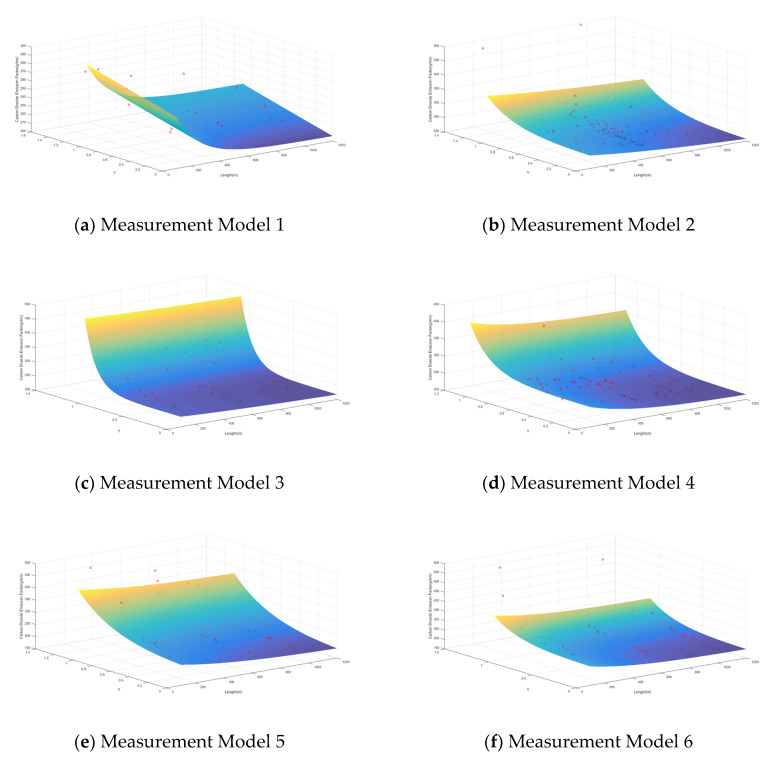

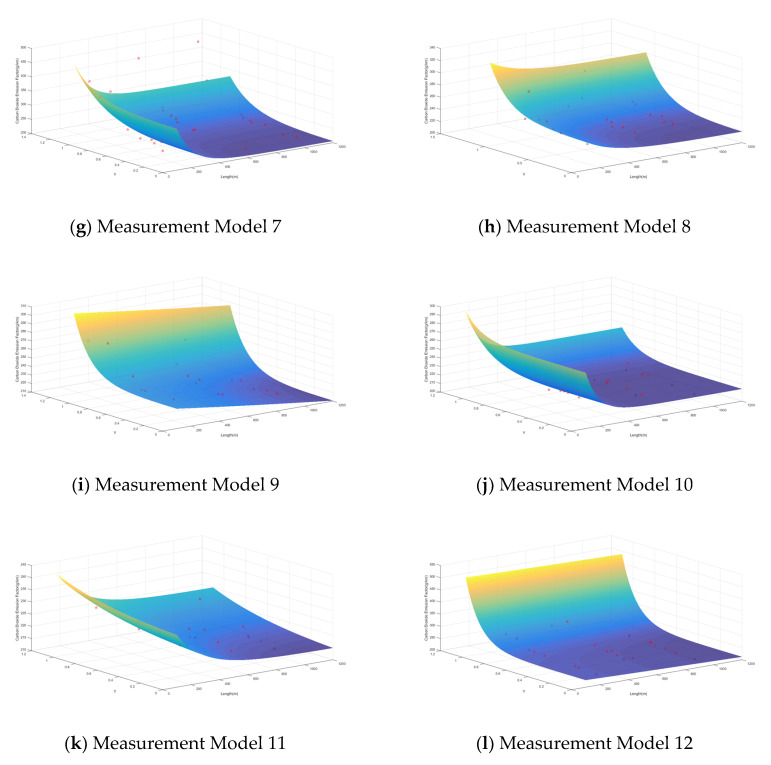
Graphics for the twelve measurement models and data used for model calibration. (**a**): Measurement Model 1, (**b**): Measurement Model 2, (**c**): Measurement Model 3, (**d**): Measurement Model 4, (**e**): Measurement Model 5, (**f**): Measurement Model 6, (**g**): Measurement Model 7, (**h**): Measurement Model 8, (**i**): Measurement Model 9, (**j**): Measurement Model 10, (**k**): Measurement Model 11, (**l**): Measurement Model 12.

The measurement models were calibrated with data from the traffic simulation and calculation of carbon dioxide emissions for the twelve types of basic road networks, which are shown in Figure 2. The results are listed in Table 2, which indicates the fulfillment of the third specific goal of the paper. It is obvious that values for parameter *m_i_* were all negative, indicating that for all the measurement models, the carbon dioxide emission factors will increase when any of the segment lengths of the road network decreases. Furthermore, values for parameter *n_i_* were all positive, indicating that for all the measurement models, the carbon dioxide emission factors will increase when any of the traffic volumes of the critical lane groups increases. R-squared statistics of all the models were bigger than 0.8, indicating a very good model fit. The twelve measurement models for the carbon dioxide emission factors and data used for model calibration are illustrated in Figure 7. The graphics of the measurement models imply the same trends as discussed above as far as the relationships between the explanatory variables and carbon dioxide emission factors are concerned.

## 7. Discussion

This paper put forth a methodology for setting up measurement models for the carbon dioxide emission factors of passenger cars for some specific types of road network in downtown areas of Chinese counties, based upon traffic simulation. As comprehensive explanatory variables have been adopted and they are compounded from various factors representing different characteristics of roads and traffic, they can be used to analyze the effects of variance concerning both traffic supply and traffic demand, resulting from schemes of urban and transportation planning or from social and economic development. The use of microscopic traffic simulation enables it to be possible to evaluate all kinds of circumstances in which vehicles have different carbon dioxide emission factors. With some adjustment, the methodology can also be used to set up measurement models for other kinds of emissions such as carbon monoxide, nitrogen oxides, and PM (particulate matter) emissions, or for other types of vehicles and roadway systems.

As for economic policies for controlling CO_2_ emissions, it has been proven that emissions taxes are effective tools for the reduction of carbon dioxide emissions [26]. Measurement models developed by the methodology of the paper can be used to analyze the results of such policies. Emissions taxes can influence people’s behavior and lead to less usage of private cars. This will bring about a decrease in the traffic volumes of road segments, resulting in smaller CO_2_ emission factors and a reduction in total CO_2_ emissions. Along with approaches for the analysis of traffic demand, the measurement models can not only be used to estimate the present CO_2_ emissions of motorized vehicles before policies have changed, but can also be used to forecast CO_2_ emissions when new policies are implemented.

The 12 measurement models developed by the paper were applied to calculate the carbon dioxide emission factors of passenger cars in the downtown areas of four typical Chinese counties during the morning peak hours. To facilitate the calculation, the traffic demands of the four counties were analyzed to obtain the traffic volumes of road segments. Results of the calculations indicated that the average CO_2_ emission factor of passenger cars for Changxing County was 242.5 g/km, the average value for Qingcheng County was 223.0 g/km, the average value for Jintang County was 212.0 g/km, and the average value for Wuan County was 202.2 g/km. By comparison, Changxing County had the biggest average CO_2_ emission factor among the four Chinese counties. This situation resulted from the more serious traffic congestions in downtown areas of Changxing County and improvement measures aiming at the alleviation of traffic congestion during rush hours should be considered.

The measurement models indicated that carbon dioxide emission factors will increase when any of the segment lengths of the road network decreases or when any of the traffic volumes of the critical lane groups increases. This conclusion is meaningful for city and transportation planning and traffic management and control. When planning a new urban area, blocks should have an appropriate size so that their bordering streets or roads shall be long enough for traffic to go through efficiently. Too short streets or roads will hamper the movement of traffic and cause more delays at intersections and therefore produce more carbon dioxide emissions. When a street or road is very short, it is better to coordinate the signalized intersections at the two ends of the segment so that efficient vehicular movements can be achieved. When vehicles are densely distributed in a small part of a road network, which is often the case with most central business districts of urban areas, traffic congestions are usually inevitable, which will lead to more carbon dioxide emissions. A more feasible solution is to encourage people to park their vehicles outside such districts and shift to other modes of transportation such as buses or bikes.

When adjacent signalized intersections are not coordinated, which is common in most urban areas of Chinese counties, a road network can be divided into a certain number of basic road networks as defined above, whose mutual relationships are usually very weak and can be ignored altogether. In such cases, it is feasible to evaluate the carbon dioxide emissions of every basic network using measurement models put forth by this paper. Then, the total carbon dioxide emissions of the whole road network can be evaluated by the simple addition of all the emissions of the basic road networks, which compose the whole road network. However, when signalized intersections are coordinated, basic road networks will interact with each other, which will result in rather complicated situations. It is therefore suggested that further research be carried out to explore the impact of signal coordination upon carbon dioxide emissions.

## 8. Conclusions

It was assumed that a road network could be divided into basic elements such as those basic road networks that have a signalized intersection and several adjacent road segments that send vehicles to the intersection. In all, basic road networks were classified into 12 types by the analysis of the characteristics of roads in the downtown areas of four Chinese counties. For any specific type of basic road network, based upon the microscopic traffic simulation with PTV Vissim, the carbon dioxide emission factors of passenger cars were calculated for various situations with different segment lengths, traffic volumes, and signal control plans using MOVES software. Altogether, 355 various schemes concerning different road networks, traffic volumes, and signal control plans were simulated and their corresponding carbon dioxide emission factors were calculated with segment length ranging from 200 to 1100 m, total traffic volumes of the intersections ranging from 417 to 5611 veh/h, and carbon dioxide emission factors ranging from 210 to 572 g/km. Based upon these data, 12 measurement models for carbon dioxide emission factors were developed for each of the twelve types of basic road networks with very good model fit.

The following limitations should be considered when similar studies are to be carried out in the future. First, the research did not consider the complex interactions between adjacent intersections. Second, the types of basic road networks defined by the paper were very limited. Some other types of basic road networks such as those with misshaped intersections should be taken into consideration in future studies. Finally, the research ignored the impact of pedestrians and bicyclists, which influences the movements of motorized vehicles when they cross roads at intersections or at other places of road segments, and may have some impact upon the carbon dioxide emissions of passenger cars.

## Figures and Tables

**Table 1:** Comparison between various measurement models for carbon dioxide emission factors.

Number	Measurement Models	R^2^	AIC	BIC
1	$CF=a\cdot L_{A}+b\cdot Y+c$	0.630	511.1857	519.7582
2	$CF=a\cdot \exp\left(m\cdot L_{A}\right)+b\cdot Y+c$	0.630	512.5316	523.2473
3	$CF=a\cdot L_{A}+b\cdot \exp\left(n\cdot Y\right)+c$	0.920	563.7208	570.1503
4	$CF=a\cdot \exp\left(m\cdot L_{A}\right)+b\cdot \exp\left(n\cdot Y\right)+c$	0.940	401.0147	413.8735
5	$CF=a\cdot \ln\left(m\cdot L_{A}\right)+b\cdot \ln\left(n\cdot Y\right)+c$	0.621	529.0839	537.6565
6	$CF=a\cdot \exp\left(m\cdot L_{A}\right)+b\cdot \ln\left(n\cdot Y\right)+c$	0.938	401.0644	411.7801

**Table 2:** Parameters of the measurement models for the carbon dioxide emission factors.

i	*a_i_*	*m_i_*	*b_i_*	*n_i_*	*c_i_*	Goodness-of-Fit (R^2^)
1	91.392	−0.006	370.847	0.065	−182.513	0.844
2	56.662	−0.001	2.652	3.265	170.788	0.935
3	27.147	−0.001	0.031	8.078	190.875	0.885
4	56.157	−0.003	0.299	5.531	194.583	0.940
5	56.877	−0.002	13.085	2.386	155.344	0.868
6	69.446	−0.003	2.678	3.384	175.643	0.916
7	315.853	−0.009	1.013	4.272	187.509	0.915
8	43.001	−0.005	0.494	4.587	199.162	0.801
9	122.963	−0.000171	0.131	5.707	92.471	0.951
10	141.137	−0.011	0.014	6.922	196.101	0.866
11	25.234	−0.005	1.629	1.735	194.891	0.831
12	104.659	−0.000148	0.047	7.812	106.460	0.975

*a_i_*, *m_i_*, *b_i_*, *n_i_*, and *c_i_* are the parameters of the ith measurement model for the ith type of road network.

## Data Availability

The data presented in this study are available on request from the corresponding author. The data are not publicly available due to the requirement of our funder.
